# Current Understanding of Molecular Pathophysiology of Heart Failure With Preserved Ejection Fraction

**DOI:** 10.3389/fphys.2022.928232

**Published:** 2022-07-07

**Authors:** Heidi Budde, Roua Hassoun, Andreas Mügge, Árpád Kovács, Nazha Hamdani

**Affiliations:** ^1^ Institut für Forschung und Lehre (IFL), Molecular and Experimental Cardiology, Ruhr University Bochum, Bochum, Germany; ^2^ Department of Cardiology, St. Josef-Hospital, Ruhr University Bochum, Bochum, Germany

**Keywords:** HFpEF, oxidative stress, comorbidities, inflammation, signaling pathways

## Abstract

Heart Failure (HF) is the most common cause of hospitalization in the Western societies. HF is a heterogeneous and complex syndrome that may result from any dysfunction of systolic or diastolic capacity. Abnormal diastolic left ventricular function with impaired relaxation and increased diastolic stiffness is characteristic of heart failure with preserved ejection fraction (HFpEF). HFpEF accounts for more than 50% of all cases of HF. The prevalence increases with age: from around 1% for those aged <55 years to >10% in those aged 70 years or over. Nearly 50% of HF patients have HFrEF and the other 50% have HFpEF/HFmrEF, mainly based on studies in hospitalized patients. The ESC Long-Term Registry, in the outpatient setting, reports that 60% have HFrEF, 24% have HFmrEF, and 16% have HFpEF. To some extent, more than 50% of HF patients are female. HFpEF is closely associated with co-morbidities, age, and gender. Epidemiological evidence suggests that HFpEF is highly represented in older obese women and proposed as ‘obese female HFpEF phenotype’. While HFrEF phenotype is more a male phenotype. In addition, metabolic abnormalities and hemodynamic perturbations in obese HFpEF patients appear to have a greater impact in women then in men (Sorimachi et al., European J of Heart Fail, 2022, 22). To date, numerous clinical trials of HFpEF treatments have produced disappointing results. This outcome suggests that a “one size fits all” approach to HFpEF may be inappropriate and supports the use of tailored, personalized therapeutic strategies with specific treatments for distinct HFpEF phenotypes. The most important mediators of diastolic stiffness are the cardiomyocytes, endothelial cells, and extracellular matrix (ECM). The complex physiological signal transduction networks that respond to the dual challenges of inflammatory and oxidative stress are major factors that promote the development of HFpEF pathologies. These signalling networks contribute to the development of the diseases. Inhibition and/or attenuation of these signalling networks also delays the onset of disease. In this review, we discuss the molecular mechanisms associated with the physiological responses to inflammation and oxidative stress and emphasize the nature of the contribution of most important cells to the development of HFpEF via increased inflammation and oxidative stress.

## 1 Introduction

Worldwide there are increasing number of patients with signs of cardiac decompensation such as dyspnea, lung congestion and ankle oedema, fatigue and exercise intolerance, and objective proof of heart failure (HF) such as increased B-type natriuretic peptide (BNP) level, while the left ventricular (LV) ejection fraction (EF) is normal (i.e., ≥50%). According to the actual HF guidelines both in Europe (McDonagh et al., 2021) and United States (Bozkurt et al., 2021) such HF phenotype is termed HF with preserved LV EF (HFpEF), in which cardiac dysfunction is primarily driven by impaired LV diastolic filling due to myocardial stiffening and ventricular-arterial uncoupling. Consequently, increased LV filling pressure promotes a backward failure and thereby leads to high left atrial pressure, increased pulmonary arterial pressure and right ventricular (RV) dysfunction. However, conventional pharmacological and device HF therapies have failed to reduce mortality and cardiovascular events in HFpEF (Bozkurt et al., 2021; McDonagh et al., 2021) HFpEF accounts for more than 50% of all cases of HF. The prevalence increases with age: from around 1% for those aged <55 years to >10% in those aged 70 years or over. Nearly 50% of HF patients have HFrEF and the other 50% have HFpEF/HFmrEF, mainly based on studies in hospitalized patients. The ESC Long-Term Registry, in the outpatient setting, reports that 60% have HFrEF, 24% have HFmrEF, and 16% have HFpEF. Over the past decades we have learned that HFpEF is a multi-organ syndrome involving the lungs, the vasculature, the kidneys, adipose tissue and skeletal muscle (Mishra and Kass, 2021). Co-morbidities are highly prevalent and similarly correlated to outcomes in both HFpEF and HF with reduced (i.e., ≤40%) LV EF (HFrEF) (Triposkiadis et al., 2016), but non-cardiovascular co-morbidities are more common in HFpEF than in HFrEF (Borlaug, 2020). HFpEF patients are predominantly elderly, women with obesity and/or type 2 diabetes mellitus, arterial hypertension, renal impairment, and atrial fibrillation.

## 2 Comorbidities and Risk Factors

Despite the enormous studies that attempted to cover the cellular and molecular basis of HFpEF development, a full understanding of its pathophysiology remains elusive. Consequently, the pharmacological management of the disease includes agents that counteract the deleterious effects of the maladaptive compensatory mechanisms, in the absence of evidence-based treatment (Roh et al., 2017).

The lack of a comprehensive and detailed understanding of the underlying mechanisms stems from the complex interplay between multiple factors that are involved in the pathophysiology of HFpEF. Such multi-factorial contribution gives rise to the heterogenic nature of disease presentation and progression among patients (Shah and Solomon, 2012). Although a LV EF of ˃49% is considered normal in HF, patients with HFpEF presented LV functional variety with an EF spectrum between 50–85% (Levy et al., 1996; Yancy et al., 2013; Ponikowski et al., 2016). HFpEF is also associated with several comorbidities and risk factors which add an additional layer of complexity to HFpEF etiology. Gender differences contribute also to the heterogeneity of patient population (Dunlay et al., 2017; Tromp et al., 2019).

### 2.1 Comorbidities

**FIGURE 1:**
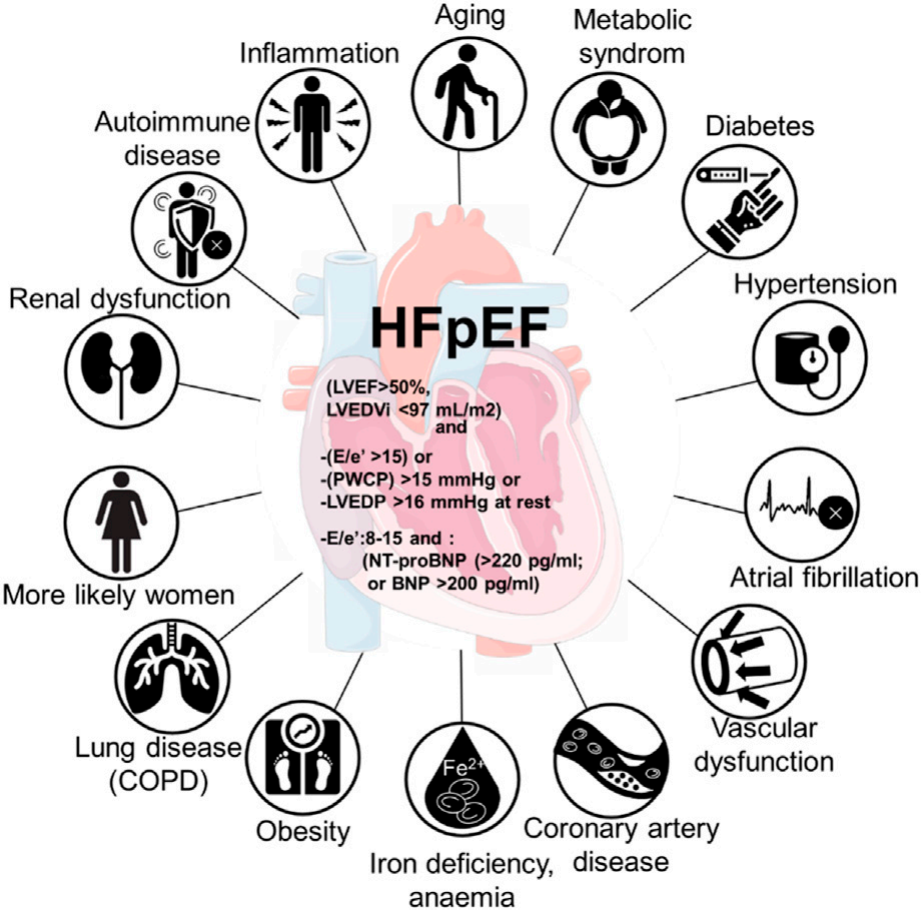
Comorbidities involved in HFpEF with the involvement of inflammation in the development of heart failure with preserved ejection fraction (HFpEF).

Several studies have reported different profiles in patients with HFrEF and HFpEF (Hogg et al., 2004; Owan et al., 2006; Fonarow et al., 2007; Zhang et al., 2019). HFpEF is characterized with higher average age and body mass index, there are more women, higher prevalence rates of atrial fibrillation and lower of ischemic heart disease. The prevalence of non-cardiac comorbidities has been reported to be higher in HFpEF patients when compared to HFrEF patients and contributing to the mortality in HFpEF patients (Shah and Gheorghiade, 2008; Riedel et al., 2018). However, there are conflicting observations. Other studies reported similar frequencies of non-cardiac comorbidities in HFpEF and HFrEF patients, comparing observational and community-based cohorts (Fu et al., 2017; Iorio et al., 2018), therefore it remains controversial whether or not the non-cardiac comorbidities differ for the outcomes in HFpEF (Felker et al., 2006; MacDonald et al., 2008; Ather et al., 2012; Smith et al., 2013; Mentz et al., 2014). In HFpEF, most prevalent comorbidities are diabetes, obesity, metabolic syndrome, chronic obstructive pulmonary disease, renal dysfunction, and anemia (Figure 1) (Mentz et al., 2014). The variety of the underlying comorbidities result in heterogenic clinical presentation of HFpEF, as well as different therapy outcomes (Lewis et al., 2017). However, it is evident that these comorbidities contribute to cardiac remodelling through systemic inflammation and microvascular damage (Paulus and Tschöpe, 2013; Franssen and González Miqueo, 2016).

Diabetes mellites is often prevalent in HFpEF patients. In addition to the systemic inflammatory state and increased oxidative stress, diabetes can contribute to the development of the disease through other pathways such as accelerating atherosclerosis, thereby leading to myocardial ischemia (Gevaert et al., 2019). The progressive renal dysfunction following diabetes promotes volume overload (Tabit et al., 2010). Hyperglycemia and insulin resistance mediate autonomic neuropathy, contributing to cardiac stiffness, hypertrophy, and fibrosis (Jia et al., 2018).

Visceral adipose tissue is associated with high levels of cytokines secretion (Berg and Scherer, 2005). Moreover, the increased plasma volume in obese patients correlates with high LV end diastolic pressure (Obokata et al., 2017). Obesity is also a potent inductor of obstructive sleep apnea, leading to chronic pulmonary vascular remodelling and pulmonary hypertension (Ayinapudi et al., 2018; Cercato and Fonseca, 2019). Of note, the co-existence of obesity and type 2 diabetes mellitus manifests in a distinct cardiometabolic phenotype of HFpEF. A recent study by Withaar et al., demonstrated the beneficial outcomes of targeting the metabolic perturbations in a multiple hit mouse model of HFpEF, which resembles the human HFpEF phenotype (Withaar et al., 2021).

Hypertension coexists with HFpEF and found to be a risk factor for increased LV afterload and pressure-induced hypertrophy. It correlated also with chronic proinflammatory state, arterial stiffness, and titin-dependent stiffness (Tam et al., 2017; Tadic et al., 2018). Furthermore, chronic arterial hypertension is known as a risk factor for renal impairment. The latter worsen the prognosis in HFpEF patients (Damman et al., 2014).

The prevalence of HFpEF increases with age. Like hypertension and diabetes, aging is linked to elevated ventricular-vascular stiffening (Redfield et al., 2005), which leads to higher prevalence of LV diastolic dysfunction with aging. Declined cellular reparative processes and oxidative stress were also shown to play important role in endothelial dysfunction during aging (Ross et al., 2016). Furthermore, age-related amyloid deposition is underdiagnosed in HFpEF with a prevalence of 13% in HFpEF patients ≥60 years old (González-López et al., 2015). Cardiac amyloidosis (CA) accounts for the treatment intolerance and low efficacy of the conventional medications such as as ACEi, ARBs, or angiotensin receptor–neprilysin inhibitor (Gallo et al., 2021), due to hemodynamic and pathophysiological features (Russo et al., 2020). It has been demonstrated that the inclusion of undiagnosed CA patients in previous trials on HFpEF such as PARAGON-HF trial corelated with poor treatment responsiveness and worse outcomes (Solomon et al., 2018). In summary, the diversity of comorbidities and risk factors manifests in distinct clinical phenotypes of HFpEF as well as different outcomes in patients (Samson et al., 2016). Hence, the proper diagnosis and evaluation of the clinical relevance of comorbidities/risk factors in patients’ sub-groups are crucial for disease management.

### 2.2 Gender differences

The incidence of HFpEF is higher in women (Duca et al., 2018), indicating that gender differences contribute to disease development. HFpEF treatment response is suggested to be gender dependent. In TOPCAT (Aldosterone Antagonist Therapy for Adults with Heart Failure and Preserved Systolic Function; NCT00094302) trial, and unlike men, women exhibited improved prognosis (Merrill et al., 2019). Gender-dependent comorbid conditions were identified in HFpEF, leading to different phenotypes and treatment response (Kao et al., 2015; Ahmad et al., 2018). Women with HFpEF are more likely to be obese and to have a history of hypertension or renal impairment. They also show higher levels of total cholesterol than males (Redfield et al., 2005; Lam et al., 2012; Westermann and Heymans, 2014). In general, women tend to show more concentric LV remodeling, with smaller LV volumes, higher EF and increased diastolic LV and arterial stiffness (Redfield et al., 2005; Lam et al., 2012; Westermann and Heymans, 2014). In contrast, men, are more likely to show an ischemic cause for HFpEF, with atrial fibrillation and chronic obstructive pulmonary disease (Redfield et al., 2005; Lam et al., 2012; Westermann and Heymans, 2014). However, others reported that atrial fibrillation increases the risk of HF hospitalization in women more than in men (Ball et al., 2013; Scheuermeyer et al., 2015; Xiong et al., 2015). The detailed mechanisms underlying the gender-related differences in HFpEF are currently not understood. Even though gender seems to play an important role for HFpEF evolution and development, only few studies have specifically addressed gender-related differences in HFpEF cohorts (Lam et al., 2012; Gori et al., 2014). Women show higher LV EF, worse diastolic function and less co-morbid conditions when compared to men (Lam et al., 2012; Gori et al., 2014). The impact of comorbidities differs also between women and men. Hypertension increases the risk of HF by 3 times in women compared to men by only 2 times (Levy et al., 1996). In addition to diabetes mellitus, which has been shown to be more evident in women with HF and increasing the HF risk twice compared to men (Kannel et al., 1974). Furthermore, atrial fibrillation increases the risk of HF hospitalization in women more than in men (O'Neal et al., 2017). Most likely because women have stronger immune responses compared to men, this could subsequently contribute to clinical outcomes between the sexes due to the distinct impact on the development of diastolic dysfunction (Klein and Flanagan, 2016). In addition, ventricular–arterial coupling (VAC) plays an important role in the physiology of cardiac and aortic mechanics, as well as in the pathophysiology of cardiac disease. The interaction between ventricular and arterial stiffness is very important as it significantly impacts the components of optimal coupling—blood pressure homeostasis and preservation of adequate cardiovascular reserve. Systemic inflammatory processes and oxidative stress may cause an increase in arterial stiffness, leading to increased myocardial stiffening (Paulus and Tschöpe, 2013). HFpEF patients have elevated vascular stiffness and increased ventricular stiffness in both systole and diastole (Kawaguchi et al., 2003; Zile et al., 2004; Melenovsky et al., 2007). Abnormal VAC is directly correlated with greater severity of HF and worse functional capacity, in addition to HFpEF showing a worse VAC than HFrEF (Pugliese et al., 2022). Therefore, ventricular-vascular stiffening can be treated acutely via modulation of ventricular systolic and diastolic performance, and endothelial function. Hence, the divergent treatment responses and HFpEF presentation could be attributed to the complex interaction between comorbid conditions, inflammation, hormones, and sex-specific gene regulation and expression (Regitz-Zagrosek et al., 2010; Beale et al., 2018; Vidal-Gómez et al., 2018).

### 2.3 Ejection Fraction

As a matter of fact, it is an ongoing question whether still EF is the adequate parameter to classify HF patients (Triposkiadis et al., 2019). On the one hand, using EF is advantageous because it is easy to understand, measure and follow non-invasively, even at bedside. On the other hand, reasonable limitations query the diagnostic role of EF in HFpEF. EF is a continuous variable in the general population that is in part attributable to age and ethnicity (), while cut-off values of a normal EF are different for males and females as well (Galderisi et al., 2017). In addition, LV EF transitions after discharge are also challenging (Dunlay et al., 2012). Important to note, HF patients with a preceding reduced LV EF (≤40%), who later show LV EF ≥50%, should be considered and treated as recovered HFrEF or ‘HF with improved LV EF’ and not HFpEF (McDonagh et al., 2021). Inter-observer and even intra-observer variabilities also bias reliability of using a single parameter to clinically describe a patient. One should keep in mind that HF is presented with both systolic and diastolic dysfunction, independent of LV EF. Accordingly, HFpEF patients show lower ventricular systolic strain, reduced systolic and diastolic longitudinal functional reserve (Triposkiadis et al., 2019)*.* Similarly, diastolic dysfunction is present in the vast majority of HF patients (Brucks et al., 2005). Finally, RV dysfunction is common in HFpEF with poor clinical prognosis (Melenovsky et al., 2014; Mohammed et al., 2014). The decline in RV function is more rapid than the decline in LV function of HFpEF patients (Obokata et al., 2019). However, despite the more rapid progression of the RV to failure, RV remodeling is apparently highly reversible (Poels et al., 2015). In conclusion, standard echocardiography in HFpEF is preferred to exclude HFrEF, dilated LV, ischemia, valvular or pulmonary diseases (Triposkiadis et al., 2019).

Patients with HFpEF exhibit LV EF variations. LV EF-based classification in HFpEF includes 3 subgroups, group 1 with LV EF ≥ 50%, group 2 with LV EF of = 60–70%, and group 3 with LV EF ˃70%. The treatment effect of spironolactone in TOPCAT trial was found to vary according to the target group (Solomon et al., 2016). Benefit of spironolactone with respect to the primary outcome and HF hospitalization was greatest in patients at the lower end of the LV EF spectrum, while no benefit observed in patients with LV EF over 65% (Solomon et al., 2016). Similarly in CHARM (The Candesartan in Heart Failure: Assessment of Reduction in Mortality and Morbidity) program, candesartan efficacy declined with higher EF (Lund et al., 2018). The study demonstrated that candesartan reduced cardiovascular death and heart failure hospitalization only at the lower end of the EF spectrum (Lund et al., 2018). Consistent with the degree of neurohumoral overactivity, it has been suggested that RAAS blockade alone may have limited benefit in HF patients with supra-normal EF (Solomon et al., 2016; Triposkiadis et al., 2019). Taken together, and in addition to comorbidities and gender differences, LV EF spectrum adds to HFpEF heterogenicity and contributes to its pathophysiology. A proper patient classification and a better understanding of comorbidities can help establishing novel evidence-based therapy for HFpEF.

## 3 Therapeutic Options in HFpEF, Between Facts and Belief

Neurohumoral upregulation and sympathetic stimulation occur in the entire HF spectrum, being modest in patients with HFpEF (Benedict et al., 1993)*.* Perhaps not surprisingly, targeting the renin-angiotensin-aldosterone system (RAAS) is highly beneficial in HFrEF, but appears to be less important in HFpEF (McDonagh et al., 2021) Perindopril (Cleland et al., 1999; Cleland et al., 2006), candesartan (Yusuf et al., 2003), irbesartan (Massie et al., 2008), spironolactone (Edelmann et al., 2013; Pitt et al., 2014) and sacubitril/valsartan (Solomon et al., 2012; Solomon et al., 2019) showed neutral outcomes in large clinical trials on HFpEF patients. Interestingly, the “neutral outcome data” showed some heterogeneity in prespecified subgroups. For example, the therapy with sacubitril/valsartan as compared to valsartan alone in the recent PARAGON HF trial (Solomon et al., 2018) revealed some potential benefit in women and those patients with midrange LV ejection fraction (Table 1).

**TABLE 1:** Summary of the clinical trials in HFpEF patients reporting the treatment outcomes of some pharmacological therapy options.

Acronym	Identifier	Participants	Intervention	Outcomes	References
PARAGON HF	NCT01920711	4,822	Sacubitril-valsartan	Potential benefit in women and those patients with midrange LV ejection fraction	Solomon et al. (2018)
RELAX	NCT00763867	216	Sildenafil	The study did not observe a significant improvement	Redfield et al. (2013)
SOCRATES PRESERVED	NCT01951638	477	Vericiguat	Failed to improve NT-proBNP blood levels, although the quality of life improved	Pieske et al. (2017)
CAPACITY-HFpEF	NCT03254485	196	Praliciguat	No effect in peak VO_2_ or echocardiographic parameters	Udelson et al. (2020)
VITALIY HFpEF	NCT03547583	789	Vericiguat	Did not improve the physical limitation score of the Kansas City Cardiomyopathy Questionnaire (KCCQ)	Armstrong et al. (2020)
DILATE-1	NCT01172756	36	Riociguat	Improved exploratory hemodynamic and echocardiographic parameters. No effect on mean pulmonary artery pressure (mPAP)	Bonderman et al. (2014)
D-HART 2	NCT02173548	31	Anakinra	Reduced CRP and NT-proBNP plasma levels. Failed to improve aerobic exercise capacity or ventilation efficiency	Van Tassell et al. (2018)
TOPCAT	NCT00094302	1767	Spironolactone	Reduction in all-cause mortality associated with spironolactone therapy in women	Merrill et al. (2019)
EMPEROR-preserved	NCT03057951	5,899	Empagliflozin	Reduced the combined risk of cardiovascular death or hospitalization regardless of the presence or absence of diabetes	Anker et al. (2021)

Furthermore, the dosage used of ACE inhibitors (ACEi) and AT1-blockers (ARB) in HFpEF patients may have an important impact. In a meta-analysis (Khan et al., 2017) higher doses of ACEi or ARB significantly though modestly improved the composite end point of all-cause mortality or HF associated hospitalizations. This finding is supported by recent meta-analysis comparing the impact of ACEi, ARB, beta-blockers and the sGC stimulator vericiguat on all-cause mortality and HF associated need for hospitalization in HFpEF patients (Lin et al., 2021). The meta-analysis included 14 randomized trials. No regime could reduce all-cause mortality, but ACEi and ARB significantly reduced HF hospitalization. Even more controversial than the inhibition of the RAAS system is the use of beta-blockers in HFpEF patients (Guazzi, 2021). Major randomized trials are lacking. The benefit with nebivolol is apparently independent of EF in elderly patients with HF (van Veldhuisen et al., 2009). In a meta-analysis including 2 randomized and 10 observational studies, the use of beta-blockers appears to associate with a lower risk of all-cause mortality in HFpEF patients (Liu et al., 2014). Similar results have been reported in another metanalysis which explored the role of the blockade of the neurohormonal systems in HFpEF (Gallo et al., 2020). However, in a propensity score matching study including 14,434 HFpEF patients, the use of beta-blockers was not associated with any change in HF hospital admissions or cardiovascular death (Meyer et al., 2021).

A hallmark in myocardial cells obtained from animal models of HFpEF (mice, rats, and dogs) with diastolic dysfunction or human heart biopsies from patients with HFpEF appears to be a reduced activity of protein kinase G (PKG), possibly because of increased inflammatory and oxidative stress (Borbély et al., 2009; Bishu et al., 2011; van Heerebeek et al., 2012; Hamdani et al., 2013a; Hamdani et al., 2013b; Hamdani et al., 2014). It is tempting to speculate that some co-morbidities observed in patients with HFpEF (such as obesity, diabetes, hypertension) may promote proinflammatory and oxidative stress signals, culminating in a weakening of the cGMP-PKG pathway in myocardial cells, and therefore contributing to myocardial stiffness. Consequently, boosting of the cGMP-PKG pathway could be a promising target to improve diastolic function in HFpEF patients (Bishu et al., 2011; Hamdani et al., 2013b; Hamdani et al., 2013c; Hamdani et al., 2014), however one may think that reducing inflammation and oxidative stress could be the most beneficial way to improve all the signaling cascades that are hallmark of HFpEF development. This idea is supported by the recent outcome of many studies given the evidence of the beneficial effects of sodium–glucose cotransporter (SGLT) 2 inhibitors on improving the diastolic function via reducing inflammation and oxidative stress thereby improving different signaling cascades among them cGMP-PKG pathway and thereby cardiomyocyte function. In rat animal model of HFpEF and HFpEF patients (Pabel et al., 2018; Kolijn et al., 2021).

The RELAX trial (Redfield et al., 2013) randomized 216 HFpEF patients to the phosphodiesterase 5 inhibitor (PDE5) sildenafil (3 × 20 mg/d up to 3 × 60 mg/d) for 24 weeks, the primary end point was exercise capacity as assessed by measuring of peak oxygen consumption. Unexpected, the study did not observe a significant improvement. Of importance, more than 100 HFpEF patients studied, sildenafil failed to raise plasma cGMP levels or ameliorate diastolic LV dysfunction (Redfield et al., 2013). However, in a pilot study with HFpEF patients and pulmonary hypertension, sildenafil significantly improved exercise capacity, pulmonary hemodynamic parameters, and RV function (Belyavskiy et al., 2020), indicating again that therapeutic strategies in HFpEF patients may fit in defined subgroups, rather than fit in all.

An alternative approach for boosting the cGMP-PKG pathway may be a therapy with the direct stimulator of the sGC vericiguat. In a phase II dose-finding study, the effect of vericiguat (1.25 up to 10 mg/d for 12 weeks) on N-terminal pro-B type natriuretic peptide (NT-proBNP) blood levels was studied in 351 patients with HFrEF (SOCRATES-REDUCED trial) (Gheorghiade et al., 2015). The treatment with vericiguat was well tolerated, and the highest dose of vericiguat used (10 mg/d) was associated with a significant reduction of NT-proBNP levels. So far, four outcomes studies in HFpEF patients treated with vericiguat have been conducted (CAPACITY HFpEF, VITALIY HFpEF, SOCRATES-PRESERVED and DILATE-1 trials). A meta-analysis has been performed by Thakker et al. (Thakker et al., 2021). Overall, the treatment with vericiguat did not improve the 6-min walk distance nor the Kansas City Cardiomyopathy Questionnaire physical limitation score. Again, it is questionable whether vericiguat in the dose range up to 10 mg/d may be effective enough to “booster” the cGMP-PKG pathway. In the SOCRATES PRESERVED trial (Pieske et al., 2017) it failed to improve NT-proBNP blood levels, although the quality of life improved. It is evident that systemic inflammation plays a major role in the pathogenesis of HF (Rauchhaus et al., 2000). The increased inflammatory immune activation was suggested to contribute to exercise intolerance in HFpEF. In Diastolic Heart Failure Anakinra Response Trial 2 (D-HART 2), IL-1 blockade by anakinra, a recombinant IL-1 receptor antagonist, reduced CRP (C-reactive protein) and NT-proBNP plasma levels in HFpEF patients after 12 weeks of treatment (Van Tassell et al., 2018), however it failed to improve aerobic exercise capacity or ventilation efficiency (Van Tassell et al., 2018). The absence of any beneficial effect of anakinra on cardiorespiratory fitness was attributed to the study limitations such as lower than expected power of the study or the very high prevalence of obesity among the patients enrolled in this trial (Van Tassell et al., 2018).

As a matter of fact, HFpEF clinical trials conducted to date have shown some important inconsistencies regarding inclusion criteria that per se implicate the complexity and clinical heterogeneity of HFpEF patients. On the other hand, non-uniform trial designs also limit the comparison of the outcomes of certain drugs/classes. For this reason, different scoring systems have been proposed to help the diagnosis (Reddy et al., 2018; Pieske et al., 2019). Nevertheless, the H2FPEF and HFA-PEFF methods differ in echocardiographic cut-off values and inclusion of biomarkers, as well as the role of co-morbidities, invasive hemodynamic assessment and exercise stress testing (Nikorowitsch et al., 2021). Accordingly, the diagnosis of HFpEF remains a challenge (McDonagh et al., 2021).

Within the clinical heterogeneity of HFpEF (Mishra and Kass, 2021), prevalence of metabolic disorders such as obesity and type 2 diabetes mellitus is increasing, thereby the metabolic trigger has started becoming the dominant etiologic factor next to arterial hypertension and atrial fibrillation. Indeed, in obese HFpEF patients RV dilatation and dysfunction are more severe, while exercise capacity is worse than in non-obese HFpEF patients, suggesting an independent obesity-related HFpEF subtype (Obokata et al., 2017). Therefore, it is likely not just a coincidence that the first breakthrough in HFpEF pharmacotherapy has been made by a member of an originally oral anti-diabetic drug class of SGLT2 inhibitors (Anker et al., 2021). Although exact mechanisms of SGLT2 inhibitors are still under investigation, direct cardiac actions (e.g., improving diastolic dysfunction) of empagliflozin have been repeatedly demonstrated in both experimental and human HFpEF (Pabel et al., 2018; Kolijn et al., 2021). Regardless of diabetes mellitus in patients with HFpEF, empagliflozin reduced the combined risk of cardiovascular death or hospitalization for HF (Anker et al., 2021). At the same time, in the EMPEROR-PRESERVED trial a trend could be observed among different EF ranges, having the highest drug effect with the lowest LV EF.

Furthermore, and as previously discussed in this review, comorbidities and risk factors are major determinants of HFpEF prognosis. Hence, HFpEF treatment must include lifestyle interventions for comorbidities and risk factors management. In HFpEF patients, exercise intolerance is a primary symptom associated with low quality of life. In the Inspiratory Muscle Training and Functional Electrical Stimulation for Treatment of HFpEF (TRAINING-HF) Trial, HFpEF patients who received inspiratory muscle training (IMT) and functional electrical stimulation (FES), exhibited an increase in peak exercise oxygen uptake and improved quality of life (Palau et al., 2019), suggesting this physical therapy intervention as potential therapy option in HFpEF. In addition to physical activity, calorie restriction diet has shown favorable effects on the quality of life in HFpEF. In a randomized clinical trial that included 100 elderly, obese HFpEF patients, the 20-weeks diet group exhibited 7% decrease in body weight associated with peak VO2 enhancement of 1.3 ml/kg/min (Kitzman et al., 2016). The combination of calorie restriction diet and exercise resulted in 10% body weight decrease and improved peak VO2 of 2.5 ml/kg/min (Kitzman et al., 2016).

## 4 The Impact of Risk Factors to Disruption of Epigenetic Programming

Functional and structural changes underlying HFpEF are partially due to the clustering of risk factors that lead to endothelial dysfunction, myocyte dysfunction, tissue fibrosis, altered cellular signaling, abnormal energetics, myocardial ischemia, systemic inflammation. Various molecular and cellular mechanisms leading to the development and progression of HFpEF and epigenetic regulation may play an importnat role in the pathogenesis of HFpEF. Different mechanisms of epigenetic modifications may relate to various HF-depended settings, i.e. cardiac hypertrophy, myocardial infarction, increase in susceptibility to ischemic injury, fetal phenotype of cardiac tissue, reduce pump function, atrial fibrillation, and sudden death, and play a controversial role. For example epigenetic modifications affecting DNA methylation (Maunakea et al., 2010), ATP-dependent chromatin remodeling (Varga-Weisz, 2005), histone modifications (Zhang et al., 2005), and microRNA (Ambros, 2001) related mechanisms are considered sufficient factors contributing to adverse cardiac remodeling and preceding cardiac dysfunction and thereby heart failire development. The impact of targeting the molecular regulating epigenetic mechanisms seems to be proming therapeutic approaches (Ambrosini et al., 2022). Despite epigenetic-based therapies of HF are promised, however, no large clinical trials supported this hypothesis and preclinical data in relation to the capabilities of DNA methyltransferase inhibitors, histone deacetylase inhibitors and miRNAs to prevent HFrEF and HfpEF development.

## 5 Oxidative Stress and Inflammation in Heart Failure

### 5.1 Oxidative Stress

Oxidative stress and inflammation are evident in the pathophysiology of HFpEF (Breitkreuz and Hamdani, 2015). The oxidative stress occurs as a result of the cellular redox imbalance, i.e., increased reactive oxygen species (ROS) production, and/or decreased antioxidants availability. Although ROS play physiological role in signalling cascades, an excessive ROS formation induce cellular and myocardial damage.

In cardiomyocytes, ROS/RNS are produced from several intracellular sources, including mitochondria, NOS, and enzymes such as xanthine oxidase, NADPH oxidase (NOX), and cytochrome p450 (Zhazykbayeva et al., 2020). Several pathways are involved in the antioxidant defence system such as the well-characterised catalases and glutathione peroxidase, superoxide dismutases (SODs), and thioredoxin, in addition to a variety of antioxidant molecules, including vitamins A, C, E, glutathione, and SH groups of intracellular proteins (Rahman, 2007). The ROS/RNS are highly reactive oxygen-/nitrogen-based chemical species, which include free radicals such as O2•-, hydroxyl radical (•OH) and peroxy radicals (ROO•), and nonradicals that can generate free radicals such as hydrogen peroxide (H_2_O_2_), nitroxyl and NO (Rahman, 2007).

ROS can directly impair contractile function by modifying proteins involved in excitation-contraction coupling and myocyte contraction. These modifications include disulphide bridges, S-glutathionylation, S-nitrosylation and tyrosine nitration and chlorination leading to changes in protein function and activity (Zhazykbayeva et al., 2020). In addition, ROS induce various hypertrophy signalling kinases and mediate apoptosis. They also activate cardiac fibroblast proliferation and activate the matrix metalloproteinases (MMPs), thereby causing ECM remodelling (Tsutsui et al., 2011). These pathological processes are involved in the development of maladaptive myocardial remodeling and HF. Furthermore, mitochondrial oxidative stress and the resulting DNA damage and metabolic dysfunction can further promote repetitive cycles of ROS generation leading to progressive cellular damage (Tsutsui et al., 2011). Additionally, ROS drive endothelial dysfunction through inducing eNOS uncoupling and the subsequent NO deficiency (explained in detail in NO-sGC-cGMP-PKG pathway).

Taken together, oxidative stress drives cardiomyocytes, endothelial cells, and ECM dysfunction. All of which are major determinants of diastolic dysfunction in HFpEF patients and animal models of HFpEF (Pabel et al., 2018; Kolijn et al., 2021).

The impaired LV filling that occurs in HFpEF patients is influenced by several factors, with ventricular stiffness playing a central role in the development of diastolic dysfunction. Conceptually, a differentiation can be made between ECM-based and cardiomyocyte-based stiffness. Studies showed, that there is an interplay between ECM and cardiomyocytes (Banerjee et al., 2006). Therefore, alterations of both compartments combined, contribute to the increased myocardial passive stiffness in HFpEF patients and animal models of HFpEF (Zile et al., 2015; Franssen and González Miqueo, 2016; Hamdani et al., 2017). Previous work by us and others has suggested that comorbidities lead to systemic inflammation and increased oxidative stress, which triggers endothelial and cardiomyocyte dysfunction (Paulus and Tschöpe, 2013; Franssen et al., 2016; Schiattarella et al., 2019). Systemic metabolic inflammation, also accompanied by an increased activity of the inducible nitric oxide synthase (iNOS) and augmented nitrosative stress, which may play an important role in the pathophysiology of obesity associated HFpEF (Franssen et al., 2016). Since increased inflammation is linked with the development of HF (Van Linthout and Tschöpe, 2017), it is not surprising that interventions that reduce inflammation are being explored as potential treatment in HF.

### 5.2 Inflammation

In parallel to oxidative stress, inflammation also has a central role in the pathogenesis of HF. Because ventricular remodeling processes are due, at least in part, to oxidative or nitrosative stress, inflammation contributes to the development and progression of HF and HFpEF (Zhazykbayeva et al., 2020). Both processes act independently and *via* a crosstalk that leads to cardiac remodeling and HF (Khaper et al., 2010). The link between disproportionately high ROS and cytokine generation is evident in the reciprocal promotion of downstream signaling pathways (Khaper et al., 2010).

Acute local inflammatory responses initially serve to combat pathogens and regenerate locally damaged tissues, as well as restore homeostasis, and are therefore a useful defense mechanism and regenerative response of the body. If the inflammatory response persists, systemic chronic inflammation develops, which can lead to cardiovascular disease (Castillo et al., 2020).

The inflammatory response can be caused by damage to the myocardium, such as after a myocardial infarction, in which adverse remodeling processes are initiated by persistent and chronic inflammation. Immune-modulated remodeling processes including autoimmune triggers or infectious such as viral infections (Riehle and Bauersachs, 2019), bacteria or parasites can also activate inflammatory reactions, with viral myocarditis mainly leading to the development of dilated cardiomyopathy and HF. In some infections, lysis of cardiomyocytes may occur, with infected cardiomyocytes releasing self-antigens and these being more commonly affected than fibroblasts. Infection leads to release of various pro-inflammatory cytokines, such as interleukin (IL)-1α, IL-6, tumor necrosis factor-alpha (TNFα), and interferon gamma (IFN-γ). In addition, monocytes are activated, which in turn initiate an apoptosis response in infected cells (Castillo et al., 2020).

Other risk factors causing chronic systemic inflammatory response are noncardiac comorbidities (Zhazykbayeva et al., 2020; Mesquita, Lin, Ibrahim), which is mainly associated with the development of HFpEF.

As a result, inflammation of microvascular endothelial cells and oxidative stress occurs. HFrEF, on the other hand, is mainly triggered by damage to the heart such as coronary artery disease, tissue necrosis from postischemic injury, toxic exposure, acute physical trauma, hemorrhage, or resuscitation (Mesquita, Lin, Ibrahim), viral myocarditis or ischemia, and is therefore associated with biomechanical cardiac stress markers such as BNP (Castillo et al., 2020). In contrast, HFpEF was found to have elevated levels of circulating systemic inflammatory biomarkers including C-reactive protein (CRP), IL-1 β, IL-6, IL-10, immunoglobulin-like transcript 6, myeloperoxidase, and TNFα, confirming the comorbidity-inflammation paradigm postulated by Paul and Zile for HFpEF (Paulus and Zile, 2021a). Accordingly, HFpEF and HFrEF have different inflammatory features, and inflammation is thought to play an associative role in HFrEF, whereas HFpEF is triggered by a combination of systemic and local inflammation (Paulus and Zile, 2021a).

During inflammation, proinflammatory cytokines are released in the renal or pulmonary vasculature due to increased arterial pressure, which has been studied in hypertensive HFpEF patients and also in animal models. Similarly, the release of proinflammatory cytokines and their increase in serum are observed in patients with obesity. Here, cytokine release is mediated by infiltration of monocytes and macrophages into the increased visceral white adipose tissue (Zhazykbayeva et al., 2020). In type 2 diabetes, systemic inflammation occurs with activation of the vascular inflammasome and glucose-mediated redox stress. In COPD and chronic kidney disease patients, tissue damage leads to mobilization of leukocyte cells, which in turn sustains systemic inflammation (Mesquita, Lin, Ibrahim).

The release of proinflammatory cytokines also leads to ROS and NOS production by macrophages and monocytes, which causes an increase in oxidative stress. This oxidative stress induces mitochondrial damage, resulting in increased ROS production. In addition, mitochondrial damage also has significant effects on energy provision processes and programmed cell death (apoptosis) (Zhazykbayeva et al., 2020).

During the inflammation process, proinflammatory signaling is triggered. A common cause associated with cormobidates is metabolic stress (metabolic load). Systemic inflammation is manifested by an increase in plasma biomarkers such as CRP (C-reactive protein), TNFα, TNFαR1 (tumor necrosis factor α receptor 1), GDF15 (growth differentiation factor 15), IL6, soluble IL1R1 (interleukin 1 receptor 1), IL1RL1 (interleukin 1 receptor like 1), and integrin subunit beta 2. These biomarkers promote systemic inflammation in HFpEF, and are therefore associated with the disease (Santhanakrishnan et al., 2012; Paulus and Tschöpe, 2013; Sanders-van Wijk et al., 2015; Shah et al., 2016).

Systemic inflammation also triggers the expression of endothelial adhesion molecules such as intercellular adhesion molecule 1 (ICAM-1), E-selectin, and vascular cell adhesion molecule 1(VCAM-1), the latter being an important regulator involved in inflammation (Turhan et al., 2005). VCAM-1 supports transendothelial migration of leukocytes and recruitment of macrophages and T cells (Kong et al., 2018). Besides, endothelial NO production is being reduced as well, which in turn leads to increased ROS.

In addition to metabolic stress, hemodynamic stress is also an important contributor to the initiation of proinflammatory processes. Hemodynamic stress is caused by arterial hypertension, while profibrotic signaling pathways are activated in addition to proinflammatory cascades, as reflected by differences in biomarkers (myocardial and circulating), such as proteins, peptides, and microRNAs (miRNAs) (Paulus and Zile, 2021b).

The signaling pathways are regulated by microRNAs, which in turn are controlled by alterations in the balance of histone acetylation (HDAC). Moreover, cardiac-specific miRNAs play an important role in cardiac remodeling processes leading to HF phenotypes. Specific HF phenotypes, e.g., HFpEF or HFrEF, might be distinguished by differences in the expression of miRNA-1, miRNA-29, miRNA133, and miRNA-208, which are due to an imbalance in regulatory mechanisms. In this context, miRNAs play a crucial role as gene regulators in the control of inflammation, as they are thought to act as signaling regulators to fine-tune inflammatory responses and prevent their uncontrolled progression (Renaud et al., 2015; Berezin, 2016; Tahamtan et al., 2018; Paulus and Zile, 2021a).

Proinflammatory signals triggered by hemodynamic stress release cytokines or chemokines that induce infiltration of the myocardium by monocytes, T/B cells, and activated macrophages. Macrophage activation leads to collagen formation and fibroblast activation, resulting in remodeling of the interstitial ECM and stiffness of the myocardium. Structural remodeling of the basal lamina includes changes in laminin isoform and increases in perlecan, nidogen, and collagen IV (Hulsmans et al., 2018; Paulus and Zile, 2021a).

### 5.3 The Direct and Indirect Effect of Oxidative Stress on Cardiomyocyte-Based Stiffness

While the ECM-based changes are likely to be the main factor in the advanced stages of the disease, the cardiomyocyte-based changes occur in the early stages of HFpEF (Franssen and González Miqueo, 2016). A common structural myocardial change is LV hypertrophy. In HFpEF patients, concentric LV hypertrophy is mostly found, in which the relative wall thickness increases (Heinzel et al., 2015). Among other things, titin is involved in the hypertrophic remodeling processes. Titin is also an important factor in modulating passive stiffness of cardiomyocytes through isoform switching, oxidative modifications and phosphorylation (Franssen and González Miqueo, 2016). Some studies show that HFpEF patients and HFpEF animals have increased expression of the stiffer N2B isoform. Others show an increased N2BA:N2B ratio (Wu et al., 2002; Borbély et al., 2009; Bull et al., 2016). However, in general, no clear trend can be identified. Consequently, post-translational modifications have a higher degree of modulation of the titin spring segment, as they can occur more rapidly in contrast to slow isoform switch (Ahmed and Lindsey, 2009). Titin can be reversibly and/or irreversibly modified by ROS/RNS, resulting in disulphide bonds, S-glutathionylation, S-nitrosylation and tyrosine nitration and chlorination (Steinberg, 2013; Breitkreuz and Hamdani, 2015). Disulfide bond formation in the N2-Bus region promotes titin stiffness. In contrast, S-glutathionylation of cysteines in titin immunoglobulin (Ig-)domains prevents the refolding of unfolded titin (Alegre-Cebollada et al., 2014). Consequently, titin-based stiffness is reduced.

Furthermore, the phosphorylation of titin plays a significant role in the modulation of passive stiffness. Not only the kinase, but also the region of phosphorylation has a major influence. While phosphorylation of the N2Bus region reduces stiffness, passive tension is increased by phosphorylation of the PEVK region. These opposite effects occur, because the negatively charged phosphate group increases the negative charge by the acidic amino acids of the N2Bus region and thus increases elasticity. The negative charge of the phosphate group, on the other hand, reduces the positive charge of the PEVK region caused by basic amino acids and decreases elasticity (Kötter et al., 2013). So far, ERK, protein kinase A (PKA), PKG and calcium/calmodulin-dependent protein kinase II (CaMKIIδ) are known to phosphorylate the N2Bus region and protein kinase Cα PKCα the PEVK region. In HFpEF patients as well as in animal models (ZSF1-obese rats and old hypertensive dogs), a hypophosphorylation of titin was detected (Hamdani et al., 2013c; Franssen and González Miqueo, 2016), which was associated with high myocardial stiffness.

## 6 Oxidative Stress-Modulated Signalling Pathways in HFpEF

**FIGURE 2:**
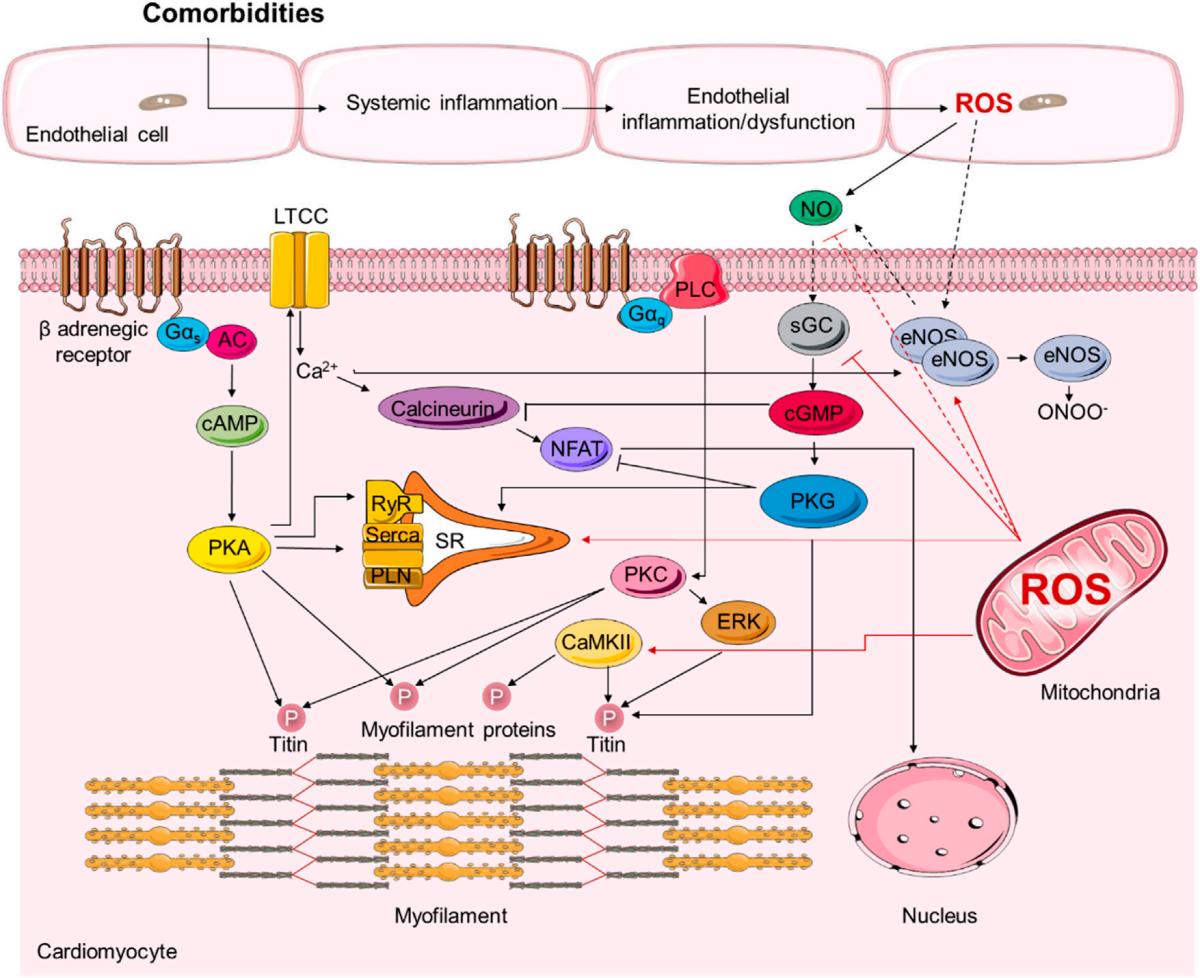
Representative scheme demonstrating the signalling pathways involved in the pathophysiology of heart failure with preserved ejection fraction (HFpEF).

It has been evident that oxidative stress and inflammation drive HFpEF development through detrimental effects on protein signalling that regulate cardiomyocyte function (Figure 2) (Zhazykbayeva et al., 2020).

### 6.1 NO-sGC-cGMP-PKG Pathway

PKG-mediated phosphorylation of titin reduces its stiffness, thereby regulating diastolic function (Linke and Hamdani, 2014). Under oxidative stress conditions, ROS uncouple eNOS which results in switching the eNOS dimer to a superoxide anion-generating monomer. The resulting RNS peroxynitrite (ONOO−) nitrates tyrosine and produces nitrotyrosine, which can further exacerbate the oxidative stress (Zhang and Shah, 2014). The decreased NO bioavailability count for endothelial and myocardial dysfunction as seen in HFpEF patients and animal models of HFpEF. The latter is promoted via the subsequent attenuation of sGC and PKG activities leading to hypophosphorylation of sarcomeric proteins, including titin, thereby increasing the myocardial stiffness, and contributing to diastolic dysfunction in HFpEF patients and animal models of HFpEF. Since the NO-cGMP pathway has been reported to supress cardiac hypertrophy through inhibiting calcineurin-NFAT signalling, its downregulation might contribute to the development of cardiomyopathies and HF (Fiedler et al., 2002). Furthermore, PKG can be directly modified by oxidative stress. PKGIα oxidation and reduced PKGIα activity were found in human HFpEF myocardium and ZDF rats and correlated significantly with increased myocardial oxidative stress (Kolijn et al., 2021). GSH supplementation could reverse the mechanical disturbances and restore the elevated F_passive_ in hypertrophic cardiomyopathy and HFpEF cardiomyocytes from patients and animal models, suggesting the oxidative stress-induced modifications in PKG pathway to play a crucial role in HFpEF and hypertrophic cardiomyopathy pathology (Hassoun et al., 2021; Kolijn et al., 2021). Moreover, we have shown empagliflozin to augment the diminished NO-sGC-cGMP-PKG pathway and enhance cardiomyocyte function due to its anti-inflammatory and antioxidant notion (Kolijn et al., 2021). These findings confirm and extend previous *in vitro* studies where incubation of myocardial preparations with active recombinant PKG increased phosphorylation of titin and reduced passive diastolic myofiber stiffness (Borbély et al., 2009; Bishu et al., 2011; van Heerebeek et al., 2012; Hamdani et al., 2013a; Hamdani et al., 2013b; Hamdani et al., 2014). Indeed, inhibition of SGLT2 has been associated with reduced inflammation and reduced activation of the nucleotide-binding domain-like 81 receptor protein 3 inflammation in liver and plasma in type 1 and 2 diabetes (Tahara et al., 2013; Gembardt et al., 2014; Tahara et al., 2014) inflammasome in the kidney (Benetti et al., 2016) and heart. In line with these thoughts and findings, improved diastolic function *in vivo* in ZDF obese rats was shown to be associated with improved inflammation and oxidative stress in both endothelial cells and cardiomyocytes (Kolijn et al., 2021). The same was true for HFpEF patients, implying for a dual effect of empagliflozin, pointing to the anti-inflammatory and anti-oxidative proprieties of this agent. It has been demonstrated that empagliflozin reduced myocardial ketone utilization and preserved glucose utilization in diabetic hypertensive heart disease (Abdurrachim et al., 2018). Others reported that empagliflozin induced a shift toward ketone body (Santos-Gallego et al., 2019), emphasizing the metabolic effect of empagliflozin. Of note, our previous study showed that comorbidities of HFpEF contribute to a systemic inflammatory state, which induces microvascular endothelial inflammation and results in endothelial dysfunction, produced ROS, reduced NO bioavailability, reduced cGMP-PKG pathway, all ultimately leading to increased cardiomyocyte stiffness as a consequence of hypophosphorylation of the giant protein titin (Hamdani et al., 2013a; Franssen et al., 2016). Empaglifozin induced a reversible shift towards pro-inflammatory cytokines and reduced oxidative stress, in turn improved endothelial and cardiomyocyte function. Cardiomyocyte stiffness is increased in HFpEF patients and animal model as reported in the current study and confirming all previous ones (Bishu et al., 2011; Hamdani et al., 2013b; Hamdani et al., 2013c; Hamdani et al., 2014; Kovács et al., 2016a). Low NO bioavailability and reduced brain-type natriuretic peptide (BNP), related to comorbidity-associated oxidative stress and inflammation, has been predicted to be the most likely underlying cause of this abnormality. Another important concept in the pathogenesis of HFpEF is that oxidative stress and inflammation, resulting from the uncoupling of NOS, leads to LV hypertrophy, fibrosis and hence diastolic dysfunction. In accordance with our hypothesis and previous findings, comorbidities raise blood levels of proinflammatory cytokines and induce cardiac microvascular inflammation. The latter alters paracrine signaling from microvascular endothelial cells to adjacent cardiomyocytes and fibroblasts: cardiomyocytes become stiff and hypertrophied due to low cGMP, and fibroblasts differentiate into myofibroblasts under the influence of transforming growth factor *ß* secreted by infiltrating macrophages. Myofibroblasts augment interstitial collagen deposition, which together with the presence of stiff cardiomyocytes, induces high diastolic LV stiffness, the main determinant of impaired exercise tolerance and symptoms in HFpEF.

Empagliflozin was previously shown to reduce diastolic tension in isolated ventricular trabecular from human failing hearts as well as in cardiomyocytes from both diabetic and non-diabetic mice (Pabel et al., 2018) via increased phosphorylation status of the myofilaments proteins cardiac troponin I (cTnI), myosin binding protein C (cMyBP-C), titin, and reducing oxidation of PKG potentially via reducing inflammatory activation and oxidative stress, thus diastolic function in HFpEF in ZDF rats. The anti-inflammatory and anti-oxidative properties of empagliflozin improves thereby also the endothelial function, likewise, the effects of empagliflozin on aortic stiffness, vascular resistance and diastolic function (Chilton et al., 2015; Verma et al., 2016; Habibi et al., 2017). Improved cardiomyocyte function is attributed mainly to increased phosphorylation of cTnI and titin dependent-PKG phosphorylation.

Increased peripheral inflammation, monocytosis and monocyte differentiation to anti-inflammatory/profibrotic M2 macrophages are associated with HFpEF, mainly patients with a high prevalence of metabolic comorbidities (Glezeva et al., 2015). Moreover, perivascular adipose tissue plays a major role in mediating vascular tone and endothelial inflammation through the interaction of perivascular adipocytes, immune cells, vascular endothelium and smooth muscle cells (Maier and Bers, 2007). These effects also mediated by reduced expression of eNOS and thus decreased NO synthesis, resulting in reduced vasorelaxation (Molica et al., 2015).

### 6.2 cAMP-PKA Pathway

Cardiomyocyte stiffness is partially determined by titin phosphorylation, not only by the downstream target of the cGMP-PKG, but also cAMP-PKA pathways. Altered PKA signalling, a downstream effector of cAMP, is involved in the progression of cardiomyopathies and HF (Saad et al., 2018). In the healthy heart and upon ß-adrenergic stimulation, the activation of cAMP-PKA pathway regulates the contractile function via its positive lusitropic effects. PKA phosphorylates several proteins involved in Ca^2+^ handling and contraction such as, L-type calcium channel (LTCC), sarco-/endoplasmic reticulum Ca^2+^-ATPase (SERCA), ryanodine receptors (RyR), cTnI, cMyBPC, and titin. PKA dependent phosphorylation of cTnI and cMyBPC is responsible for myofilament Ca^2+^ desensitization and the subsequent Ca^2+^ reuptake into the sarcoplasmic reticulum (Metzger and Westfall, 2004). Reduced PKA activity was reported in end stage failing human hearts and correlated with elevated oxidative stress and inflammation (Budde et al., 2021; Kolijn et al., 2021). It has been well established that PKA-dependent hypophosphorylation of myofilament proteins leads to increased myofilament Ca^2+^ sensitivity and the leakage of Ca^2+^ from the SR, which increases cytosolic calcium levels during diastole (Hamdani et al., 2013c; Herwig et al., 2020; Kolijn et al., 2021). Moreover, titin is phosphorylated by PKA at N2B spring element (Kötter et al., 2013), hence, impaired PKA signalling has also been observed in HFpEF which contributed to titin phosphorylation deficit and altered F_passive_ in HFpEF (Hamdani et al., 2013c). These pathological alterations associate with the development of cardiac dysfunction and HF. In human HFpEF biopsies and HFpEF rat model, the high F_passive_, reduced titin phosphorylation also with low PKA activity level, this downregulation was significantly improved by sGC treatment and accompanied by a reduction in pro-inflammatory cytokines and oxidative stress markers (Kolijn et al., 2020).

### 6.3 PKC and CaMKII Signalling

PKC plays multiple roles in the regulation of cardiac function. PKC-α mediates contractility, cell growth, and cardiac hypertrophy and was shown to be upregulated in HF (Dorn and Force, 2005; Hamdani et al., 2013b). Previous studies suggested the cross talk between PKC activation, MAPK, and calcineurin signalling to play an important role in the hypertrophic cardiac remodelling and HF (De Windt et al., 2000). In HF, PKC-α dependent hyperphosphorylation of titin at the PEVK region contributes, to some extent, to the myocyte stiffness and cardiac relaxation (Redfield et al., 2013; Kovács et al., 2016b). However, PEVK region is also targeted by CaMKII, which showed increased expression/activity in failing human hearts and in animal models of cardiac hypertrophy (Anderson et al., 2011; Hamdani et al., 2013d). CaMKII signalling is involved in apoptosis, hypertrophy, Ca^2+^ handling regulation, and proinflammatory signalling (Singh et al., 2009; Anderson et al., 2011). In the heart the CaMKIIδ isoform is predominant, although a CaMKIIδC splice variant is found in the cytosol (Maier and Bers, 2007) and an isoform of CaMKIIγ is also expressed (Colomer et al., 2003). Isoforms of CaMKII display a wide range of functions in the heart. CaMKII influences myocyte function by phosphorylating ion channels and Ca^2+^-handling proteins, and by modulating transcriptional activity (Colomer et al., 2003; Backs et al., 2006; Toischer et al., 2010; Anderson et al., 2011; Swaminathan et al., 2012). Phosphorylation of histone deacetylases by CaMKII results in cardiac hypertrophic growth (Backs et al., 2006). CaMKII isoforms show increased expression/activity both in failing human hearts and in animal models of cardiac hypertrophy (Anderson et al., 2011; Hamdani et al., 2013d) Furthermore, overexpression of CaMKIIδC in mouse myocardium is associated with massive cardiac hypertrophy and induces dilated cardiomyopathy and premature death, whereas knockout of the CaMKIIδ-isoform in mice attenuates pathological cardiac hypertrophy and remodelling in response to pressure overload (Backs et al., 2009; Ling et al., 2009). Inhibition of CaMKIIδ improves cardiac function and delays the onset of maladaptive remodelling (Ai et al., 2005; Sossalla et al., 2010), suggesting that CaMKII inhibition may be a therapeutic option in the treatment of chronic overload-induced heart disease (Maier and Bers, 2007; Anderson et al., 2011).

At the cardiac myofilament level, CaMKII phosphorylates cMyBP-C (Gautel et al., 1995) and cTnI (Solaro and van der Velden, 2010), alters the rates of force development and relaxation, and modulates calcium sensitivity (Hamdani et al., 2013a). While phosphorylation of titin by CaMKIIδ may benefit diastolic function, deranged CaMKII-dependent titin phosphorylation occurs in end-stage human HF and may contribute to the disease phenotype (Hamdani et al., 2013b). In another context, CaMKII is known as a promoter of cardiac hypertrophy and inflammation, processes consistently activated by myocardial injury, and which promote cardiomyopathy and lead to systolic dysfunction.

Also, CaMKII phosphorylates titin at multiple sites within the N2-Bus and PEVK spring elements thereby contributing to the regulation of titin-based myocyte stiffness. CaMKIIδ-dependent titin phosphorylation reduces F_passive_. However, and due to the high distribution of CaMKIIδ at the Z-disc region, previous reports demonstrated CaMKIIδ-dependent phosphorylation of N2-Bus to be the dominant process (Hidalgo et al., 2013). CaMKII is highly activated by ROS leading to detrimental effects on cardiac function (Erickson et al., 2008). Given that hypertrophy and inflammation are related to neurohumoral and redox signaling processes that uncouple CaMKII activation from Ca^2+^/calmodulin dependence, CaMKII may thus act as a nodal point for integrating hypertrophic and inflammatory signalling in myocardium. In addition, inhibition of CaMKII is known to improve myocardial contractility in human HF. Therefore, understanding the contribution of oxidative stress and inflammation to increased CaMKII activity, and vice versa is needed at this stage to find the right treatment options for HFpEF patients. A conclusion that was supported by the beneficial effects of empagliflozin in improving hypertrophic pathways mediated by CaMKII, PKC, and ERK2 (Kolijn et al., 2021).

## 7 Ventricular Vascular Uncoupling

In addition to structural changes in ECM and cardiomyocytes, blood vessels are also involved in the pathophysiology of HFpEF. In addition to LV stiffness HFpEF patients show increased stiffness in the blood vessels (e.g. aorta) as well (Borlaug and Kass, 2008; Gevaert et al., 2019). Thereby an interaction between cardiac function and the arterial system takes place, which is called ventricular-arterial coupling. This provides information about cardiovascular efficiency or cardiovascular performance (Gevaert et al., 2019). Ventricular-arterial coupling is the ratio of effective arterial elasticity (EA) to LV end-systolic elasticity (EES) (Ikonomidis et al., 2019). Thereby, EA defines the afterload (vascular stiffness) and EES describes LV ventricular output relatively independent of load (ventricular systolic stiffness) (Milani et al., 2006; Borlaug and Kass, 2008; Antonini-Canterin et al., 2013).

This EA/EEs ratio is reduced in HFpEF and hypertensive patients compared to a young control group (Ikonomidis et al., 2019). However, this reduced value is in a range where work and efficiency are not affected (Borlaug and Kass, 2008). It is thus disadvantageous to consider only the EA/EES ratio in HFpEF patients, as this value often indicates normal values, although the individual parameters EA and EES are increased compared to the control group. In addition, the EES parameter sometimes increases disproportionately (Kawaguchi et al., 2003; Borlaug and Kass, 2008; Ikonomidis et al., 2019). Therefore, it is better to look at the individual parameters to study the ventricular-arterial coupling. An increase in EA and EES makes systolic pressures more sensitive to changes in LV end-diastolic volume and thus more sensitive to changes in central blood volume (Borlaug and Kass, 2008). Because of systolic ventricular and arterial stiffening, small changes in blood volume lead to large effects on arterial pressure and cardiac workload (Hundley et al., 2001).

The interaction of LV systolic stiffening and arterial stiffening increases therefore systolic workload, prolongs relaxation, impairs filling and increases end-diastolic pressure (EDP), which in turn impairs diastole (Hundley et al., 2001). Besides the stiffness, the reduced aortic distensibility and arterial stiffness in HFpEF patients is above age-related levels (Hundley et al., 2001; Reddy et al., 2017). Arterial hypertension and other comorbidities impair ventricular-arterial coupling in HFpEF, which is associated with inflammatory and mechanical overload. Therefore, alteration of ventricular-arterial coupling is also considered a primary mechanism contributing to worsening HF (Ikonomidis et al., 2019).

## 8 Extracellular Matrix-Based Stiffness

In general, the ECM is not only important for structural support and maintenance, but also influences cardiac homeostasis and is responsible for force transmission and transducing key signals to cardiomyocytes (Frangogiannis, 2019). Under normal conditions, the ECM contributes to passive stiffness to prevent overstretching and tissue deformation (Kovács et al., 2016a). However, if changes in interstitial collagen properties, such as certain types of fibrillar collagen, concentration and cross-linking occur during myocardial stress, the myocardium becomes stiffer and ventricular diastolic dysfunction develop (Brower et al., 2006) The main component of the ECM is fibrillar, stiff collagen, which can be distinguished between collagen type I and III (Cimiotti et al., 2021). In patients with HFpEF, changes in the synthesis and degradation of collagen have been found, where type I collagen is accumulated, and the amount of type III collagen remain unchanged. Thus, these patients have an increased ratio of type I/III collagen (Cimiotti et al., 2021). These abnormal accumulation of fibrillar collagens leads to the remodeling of the ECM and to Fibrosis (Wight and Potter-Perigo, 2011). Furthermore, the amount of cross-linked collagen also provides information about diastolic relaxation and LV filling pressure. In HFpEF patients, parameters of diastolic dysfunction correlate with the amount of collagen cross-linking and Lysyl oxidase-like 2 (LOXL2) expression, as this enzyme catalyzes collagen cross-linking (Yang et al., 2016). The composition and structure of ECM is also influenced by collagen-producing myofibroblasts. Due to increased myocardial inflammation in HFpEF patients, fibroblasts are getting activated by circulating inflammatory cytokines and the differentiation into myofibroblasts is primarily driven by transforming growth factor *ß* (TGF-β). This promotes furthermore the development of fibrosis (Westermann et al., 2011; Wight and Potter-Perigo, 2011). In addition, collagen secretion is influenced by direct stimulation of myofibroblasts by angiotensin II and aldosterone. Another class of proteins that play an important role in collagen metabolism are the matrix metalloproteinases (MMPs). The reduced matrix degradation associated with HFpEF is caused by the suppression of MMPs or by inhibitors of metalloproteinases (TIMPs) (Cunningham et al., 2020). In summary, changes in ECM synthesis/degradation provide information on the progression of HFpEF and the underlying mechanistic pathway is a starting point for therapeutic approach to reduce LV myocardial stiffness in HFpEF patients (Spinale and Zile, 2013).

## 9 Conclusion and Future Perspective

The present review provides an overview about the current knowledge of HFpEF pathophysiology. As previously described, the guideline-directed medical therapy is the only existing option for disease management (Roh et al., 2017). Better understanding of HFpEF pathophysiology is crucial for establishing evidence-based therapies individually for each patient. However, novel research has emerged in recent years demonstrating oxidative stress and inflammation as potential mechanisms contributing to HFpEF pathology (Breitkreuz and Hamdani, 2015; Franssen et al., 2016; Zhazykbayeva et al., 2020). Cardiac and extracardiac comorbidities are shown to promote inflammation and oxidative stress which are major determinants of myocardial, endothelial, and ECM perturbations (Breitkreuz and Hamdani, 2015). Therefore, management of comorbidities and reducing oxidative stress and inflammation have a major impact on the clinical presentation and disease outcomes. Consistent with this aspect, empagliflozin showed enhanced cardiomyocyte function due to its anti-inflammatory and antioxidant notion (Pabel et al., 2018; Kolijn et al., 2021). Taken together, in addition to the comprehensive understanding of the molecular basis of HFpEF, targeting oxidative stress and inflammation, management of risk factors, determining and following risk groups are essential for a better disease prognostication and management.
